# Complete Genome Sequence of Halomonas olivaria, a Moderately Halophilic Bacterium Isolated from Olive Processing Effluents, Obtained by Nanopore Sequencing

**DOI:** 10.1128/MRA.00144-19

**Published:** 2019-05-02

**Authors:** Shohei Nagata, Konosuke M. Ii, Tomoya Tsukimi, Masahiro C. Miura, Josephine Galipon, Kazuharu Arakawa

**Affiliations:** a Institute for Advanced Biosciences, Keio University, Tsuruoka, Japan; b Systems Biology Program, Graduate School of Media and Governance, Keio University, Fujisawa, Japan; c Faculty of Environment and Information Studies, Keio University, Fujisawa, Japan; University of Maryland School of Medicine

## Abstract

Here, we report the complete genome sequence of Halomonas olivaria strain TYRC17, a moderately halophilic, Gram-negative bacterium that was isolated from olive processing effluents. The 5-Mbp genome consists of 7,375 protein-coding sequences, including a variety of genes involved in tyrosol metabolism, nitrate respiration, and the production of polysaccharides.

## ANNOUNCEMENT

*Halomonas* is a genus of Gram-negative halophilic bacteria widely distributed in high-salinity environments, and its members typically require a high NaCl concentration and alkaline pH for growth (1). Many species within the genus are known to have potential applications in biotechnology due to their capacity to produce osmoprotectants, as well as certain enzymes or exopolysaccharides, and for their role in the degradation of pollutants (1–3). A strain isolated from salted olive processing effluents, Halomonas olivaria, was shown to completely degrade tyrosol, a toxic compound found in such effluents (4, 5).

*H. olivaria* strain TYRC17, from the Mediterranean Institute of Oceanography (Marseille, France) and also available from the Leibniz Institute DSMZ–German Collection of Microorganisms and Cell Cultures (Braunschweig, Germany), was grown as described previously (4). When the optical density (OD) reached 0.3, the culture solution was centrifuged at 5,000 × *g* for 10 min at 20°C to pellet the cells. Genomic DNA was extracted and purified with the Genomic-tip 20/G kit following the manufacturer’s instructions (Qiagen, Hilden, Germany). The sequencing libraries were prepared using a rapid barcoding kit (SQK-RAB004) and sequenced in an R9.4 flow cell (FLO-MIN106) with the GridION device (Oxford Nanopore Technologies).

A total of 1,000,874 reads were obtained, with lengths ranging from 5 to 284,063 bp. After filtering the reads to retain those greater than 50,000 bp to bring coverage to just below 100×, filtered reads were assembled using Canu version 1.8 (6), using the default parameters for Nanopore data. The genome was assembled into a single contig and was manually circularized by deleting an overlapping end. An improved consensus sequence for the assembly was obtained with Nanopolish software version 0.10.2 (7), using the methylation-aware option. Genome completeness was estimated using Benchmarking Universal Single-Copy Orthologs (BUSCO) software version 1 (8) with the bacterial data set on the gVolante Web server (9). Of the 40 BUSCO group genes, 95% were completely (85%) or partially (10%) recovered in the polished assembly, indicating a certain amount of uncorrected indels. The comparison of six 16S rRNA genes in our genome with the known partial 16S rRNA sequence (1,504 bp) indicated 99.22 ± 0.12% identity. Genes were annotated using the DDBJ Fast Annotation and Submission Tool (DFAST) pipeline (10). The estimated genome size is 4,998,083 bp, with a G+C content of 55.3%. The genome was predicted to contain 7,375 putative coding sequences, 18 rRNAs, 59 tRNAs, and 1 CRISPR sequence.

In our analysis, four nitroreductases (Cluster of Orthologous Groups 0778 [COG0778]), one nitrous oxidase accessory protein (COG3420), and one P_II_ nitrogen regulatory protein (COG0347) were found. In addition, concerning sugar metabolism, 40 genes corresponding to ABC-type sugar transport systems (COG1129, COG1175, COG1879, and COG3839) were annotated. Finally, among the tyrosol metabolic pathways shown in reference 4, a gene coding for homogentisate 1,2-dioxygenase and partial sequences associated with aryl-alcohol dehydrogenation or 4-hydroxyphenylacetic acid degradation were detected. This information will be useful to reveal the mechanism of tyrosol degradation mediated by *H. olivaria* and to contribute to the field of bioremediation.

### Data availability.

The complete genome sequence of *H. olivaria* has been deposited in DDBJ under the accession number AP019416 and in the Sequence Read Archive (SRA) under the accession number PRJNA521098.
